# Interstitial *Arabidopsis*-Type Telomeric Repeats in Asteraceae

**DOI:** 10.3390/plants10122794

**Published:** 2021-12-17

**Authors:** Alexis J. Maravilla, Marcela Rosato, Inés Álvarez, Gonzalo Nieto Feliner, Josep A. Rosselló

**Affiliations:** 1 Jardín Botánico, Instituto Cavanilles de Biodiversidad y Biología Evolutiva, Universitat de València, c/Quart 80, E-46008 Valencia, Spain; maravil3@alumni.uv.es (A.J.M.); marcela.rosato@uv.es (M.R.); 2 Real Jardín Botánico (RJB), Consejo Superior de Investigaciones Científicas (CSIC), Plaza de Murillo 2, E-28014 Madrid, Spain; ines@rjb.csic.es (I.Á.); nieto@rjb.csic.es (G.N.F.)

**Keywords:** interstitial telomeric repeats, *Arabidopsis*-type ITR, FISH, Asterales, Asteraceae, cytogenetic evolution

## Abstract

Tandem repeats of telomeric-like motifs at intra-chromosomal regions, known as interstitial telomeric repeats (ITR), have drawn attention as potential markers of structural changes, which might convey information about evolutionary relationships if preserved through time. Building on our previous work that reported outstanding ITR polymorphisms in the genus *Anacyclus*, we undertook a survey across 132 Asteraceae species, focusing on the six most speciose subfamilies and considering all the ITR data published to date. The goal was to assess whether the presence, site number, and chromosomal location of ITRs convey any phylogenetic signal. We conducted fluorescent in situ hybridization (FISH) using an *Arabidopsis*-type telomeric sequence as a probe on karyotypes obtained from mitotic chromosomes. FISH signals of ITR sites were detected in species of subfamilies Asteroideae, Carduoideae, Cichorioideae, Gymnarhenoideae, and Mutisioideae, but not in Barnadesioideae. Although six small subfamilies have not yet been sampled, altogether, our results suggest that the dynamics of ITR formation in Asteraceae cannot accurately trace the complex karyological evolution that occurred since the early diversification of this family. Thus, ITRs do not convey a reliable signal at deep or shallow phylogenetic levels and cannot help to delimitate taxonomic categories, a conclusion that might also hold for other important families such as Fabaceae.

## 1. Introduction

Telomeres are the natural ends of eukaryotic linear chromosomes. They are constituted by ribonucleoprotein complexes that differ notably from other DNA sequences in both structure and function. Capping the chromosome ends by telomere structures facilitates the protection of genetic material against double-stranded breaks, degradation, and end-to-end fusion with other chromosomes, which lead to genome instability [1,2,3]. 

Telomeric regions are constituted by large stretches of DNA sequences that are GC rich and are usually tandemly arranged to attain up to several thousands of base pairs in length [4]. Overall, telomeric sequences are highly conserved across groups of unrelated organisms although lineage specific motifs are known too [5,6]. In land plants, the dominant consensus telomere repeat is composed of seven nucleotides known as the *Arabidopsis*-type (TTTAGGG)_n_ [6,7,8], but divergent repeat motifs have been recognized [9,10,11,12,13,14,15,16,17]. It has been recently suggested that beyond the telomere motif exceptions detected, the real diversity in telomeric sequences in land plants has been probably underestimated [18]. 

Tandem repeats of telomeric-like motifs have been reported also at intrachromosomal regions, known as interstitial telomeric repeats (ITR) or interstitial telomeric sequences [19]. The use of molecular cytogenetic techniques has allowed the detection of ITRs in unrelated organisms [7,8,20,21,22,23,24]. Overall, these studies have interpreted the occurrence of ITRs as stable karyological landmarks that they may be remnants of end-to-end fusion events between non-homologous chromosomes, translocations, inversions, and heterologous chromosomal recombination [19,25]. Compared to other popular landmarks used in plant molecular cytogenetics, such as the 35S and 5S rDNA families [26], efforts devoted to assess the presence, phylogenetic distribution, and evolutionary significance of ITRs in land plants have been scanty. 

Asteraceae (Compositae), with an estimated 25,000–35,000 species, comprises about 10% of all flowering plant diversity [27]. This family has been extensively characterized from a basic cytogenetic perspective, based partly on a vast amount of data accumulated on chromosome numbers [28,29,30]. This has allowed to substantiate a hypothesis concerning chromosomal base number evolution [29] and to understand the patterns of karyological change after multiple polyploidization events that have occurred across Asteraceae in its early evolutionary history [31]. 

Reports indicating the presence of ITRs in this family are known since the work of [8]. However, the number of taxa analyzed to date remains limited and includes only 53 species (and one subspecies) belonging to 20 genera [8,32,33,34,35,36,37,38,39,40,41,42,43,44,45,46,47,48,49,50]. Most of the species analyzed to date are restricted to subfamily Asteroideae (Table S1). ITRs have been detected in 12 species, six of which belong in the genus *Anacyclus* [49]. The fact that most surveys usually include sparse and limited taxonomic samplings precludes obtaining a broad perspective on the evolutionary significance of the presence, location and amplification of ITR sites in Asteraceae. 

In this paper, we assess the presence of ITRs in Asteraceae using a comparatively large sampling scheme compared to previous studies [8,32,33,34,35,36,37,38,39,40,41,42,43,44,45,46,47,48,49,50]. We aimed to cover a broad phylogenetic range and analyze the available data from an evolutionary perspective. To this end, we have used an *Arabidopsis*-type telomeric sequence as a probe to reveal the presence of ITRs in karyotypes obtained from mitotic chromosomes. In situ hybridization techniques have become one of the most powerful approaches for mapping specific sequences of DNA in plant cytogenetics, including telomere sequences [51]. Specifically, fluorescent in situ hybridization (FISH) involves the indirect (through haptens) or direct labelling of a fluorescence probe followed by annealing to the target sequences of individual cells with subsequent visualization by epifluorescence microscopy [52]. 

The overall objective was to explore whether the presence of ITRs, the site number, and their chromosomal location in Asteraceae convey any phylogenetic signal, i.e., if they were retained over evolutionary time or, alternatively, whether ITRs are labile chromosomal landmarks and thus poor phylogenetic predictors. Specifically, the goals of our study were (a) to investigate whether ITR loci are present within the most speciose subfamilies (Barnadesioideae, Mutisioideae, Carduoideae, Cichorioideae, and Asteroideae) and the monospecific Gymnarhenoideae by analysing the somatic karyotypes of 132 species and two subspecies from 108 genera and reviewing previous available data, (b) to examine the patterns of site number and chromosomal ITR distribution across major evolutionary groups, (c) to examine the association between the patterns of chromosome evolution and eventual cycles of amplification, genomic spread, and contraction of ITRs.

## 2. Results

### 2.1. New Observations

**Figure 1:**
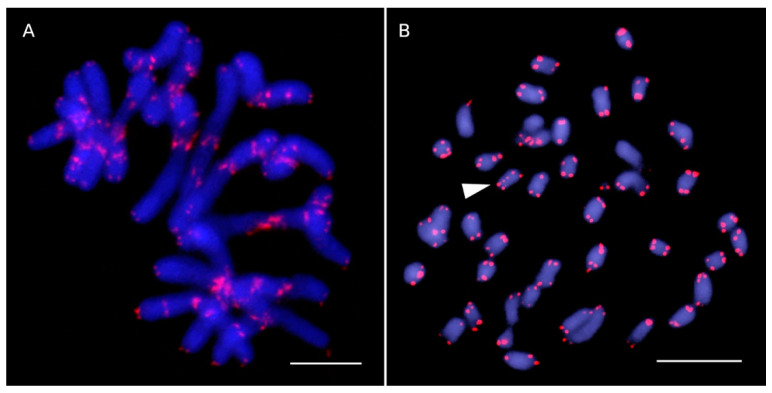
Telomeric-like sequence (TTTAGGG)_n_ sites in Asteroideae species assessed by FISH analysis. Telomeric sites and interstitial telomeric repeat (ITR) sites are shown as red fluorescent signals and the chromosomes are counterstained with 4, 6-diamidino-2-phenylindole (DAPI) (blue colour). (**A**) *Cota nigellifolia* (2*n* = 18), showing 52 ITR sites. (**B**) *Senecio vulgaris* (2*n* = 40,) with two ITR sites (arrowhead) located in the same chromosome. Scale bars: 10 µm.

**Figure 2:**
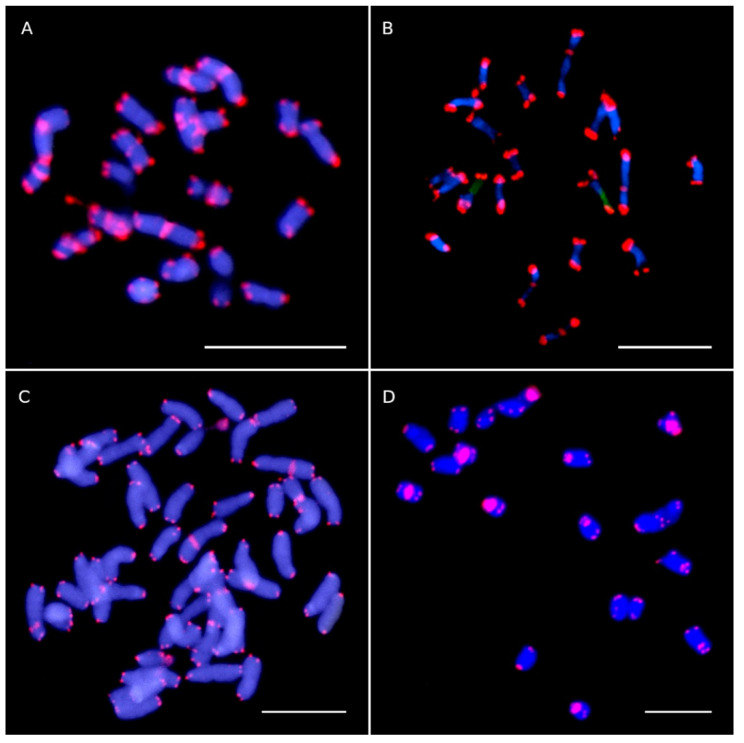
Telomeric-like sequence (TTTAGGG)_n_ sites in representative species of Carduoideae (**A**,**B**), Mutisioideae (**C**) and Gymnarhenoideae (**D**) tribes assessed by FISH analysis. Telomeric sites and interstitial telomeric repeat (ITR) sites are shown as red fluorescent signals and the chromosomes are counterstained with 4, 6-diamidino-2-phenylindole (DAPI) (blue colour). (**A**) *Galactites tomentosa* (2*n* = 20) showing 10 ITR sites. (**B**) *Carlina hispanica* (2*n* = 18 + 2B) with six ITR sites. (**C**) *Nassauvia sprengelioides* (2*n* = 44) with four ITR sites). (**D**) *Gymnarrhena micrantha* (2*n* = 20) showing ten ITR sites. Faint ITR signals are marked with arrowheads. Scale bars: 10 µm.

**Figure 3:**
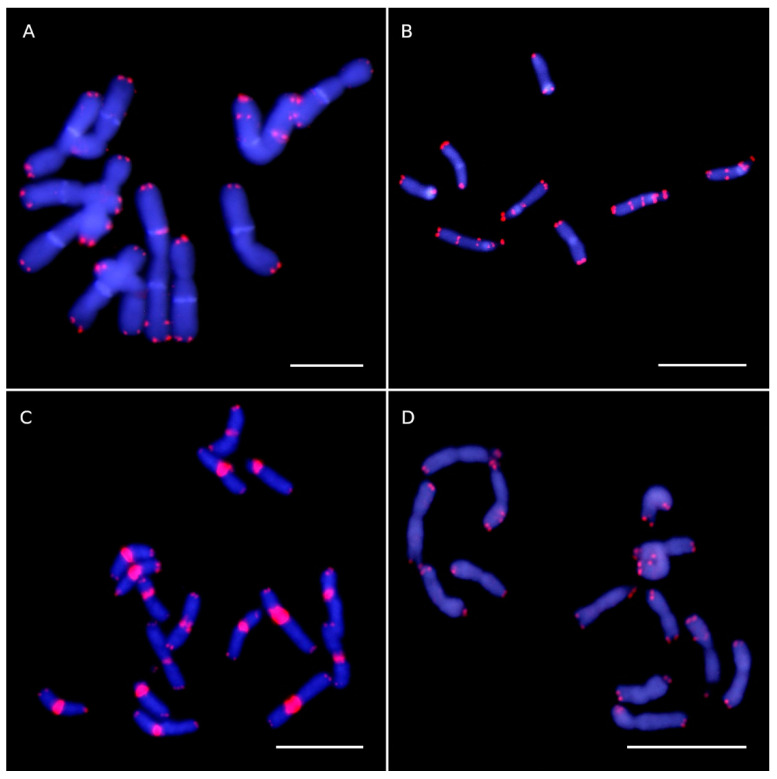
Telomeric-like sequence (TTTAGGG)_n_ sites in representative species of Cichorioideae (**A**–**D**) tribes assessed by FISH analysis. Telomeric sites and interstitial telomeric repeat (ITR) sites are shown as red fluorescent signals and the chromosomes are counterstained with 4, 6-diamidino-2-phenylindole (DAPI) (blue colour). (**A**) *Stokesia laevis* (2*n* = 14) with four sites. Two chromosomes were missed from the metaphase plate. (**B**) *Leontodon longirostris* (2*n* = 8) with ten ITR sites. (**C**) *Hyoseris taurina* (2*n* = 16) with 18 ITR sites. (**D**) *Sonchus tenerrimus* subsp. *tenerrimus* (2*n* = 14) showing two sites. Scale bars: 10 µm.

FISH signals of ITR sites were detected in species of Asteroideae (Figure 1), Carduoideae (Figure 2A,B), Cichorioideae (Figure 3), Gymnarhenoideae (Figure 2D), and Mutisioideae (Figure 2C). Interstitial telomeric-like repeats were neither observed in Barnadesioideae accessions nor in the two sampled species of the related families Calyceraceae and Goodeniaceae (Asterales) used for comparative purposes. Overall, ITR signals were observed in 24 species (18.18% of our samples) belonging to 20 genera (18.52%). 

In the studied accessions showing ITRs, the number of sites ranged from two (*Anthemis cotula*, *Tanacetum vulgare*, *Leucanthemum graminifolium*, *Senecio vulgaris*, *Dymondia margaretae*, *Sonchus tenerrimus* subsp. *tenerrimus*, and *Leontodon tuberosus*) to 52 in *Cota nigellifolia*, the highest number so far reported in Asteraceae. Intrageneric differences in the presence or absence of ITR signals were observed in *Achillea* (two absences, one presence), *Anthemis* (three absences, two presences), *Cladanthus* (one absence, one presence), *Nassauvia* (one absence, one presence), and *Sonchus* (one absence, one presence) accessions. Intraspecific variation was detected in a single species, *Sonchus tenerrimus*. Two ITR sites were present in subsp. *tenerrimus* whereas none were found in the closely related subsp. *dianae*.

**Figure 4:**
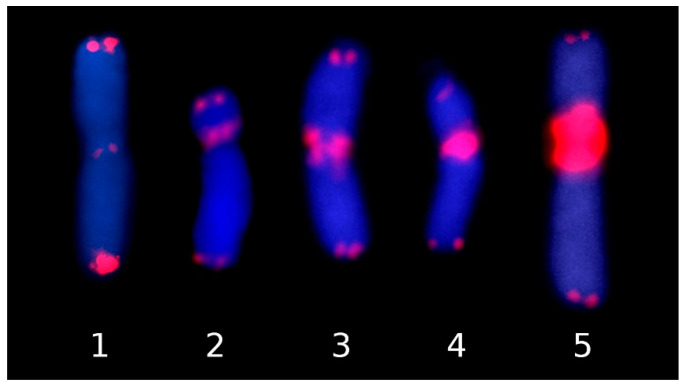
Differences in the signal intensity of ITR sites detected in Asteraceae chromosomes. (**1**): *Anacyclus clavatus*; (**2**): *Leontodon tuberosus*; (**3**–**5**): *Hyoseris taurina*.

ITR sites preferentially occur as homozygous loci (21 species). However, odd numbers of ITR sites reflecting a hemyzygous state were detected in *Gonospermum fruticosum* (29 sites), *Achillea ligustica* (17 sites), and *Anthemis tinctoria* (3 sites). The intensity of the ITR signals detected by FISH varied between site locations and samples, suggesting that a variable number of repeats are involved (Figure 4). For instance, faint ITR signals (showing lower intensity than the telomeric sites) were detected in *Stokesia laevis* (Figure 3A). The opposite was detected in *Hyoseris taurina* where the centromeric ITR sites showed a remarkably higher intensity than those present at the chromosome ends (Figure 3C).

### 2.2. Patterns of ITR Variation in Asteraceae

Our results in addition to previous reports of ITR sites in Asteraceae [8,32,33,34,35,36,37,38,39,40,41,42,43,44,45,46,47,48,49,50], including the chromosome number, presence and number of ITR signals, and their chromosomal distribution are indicated in Table S1. 

**Figure 5:**
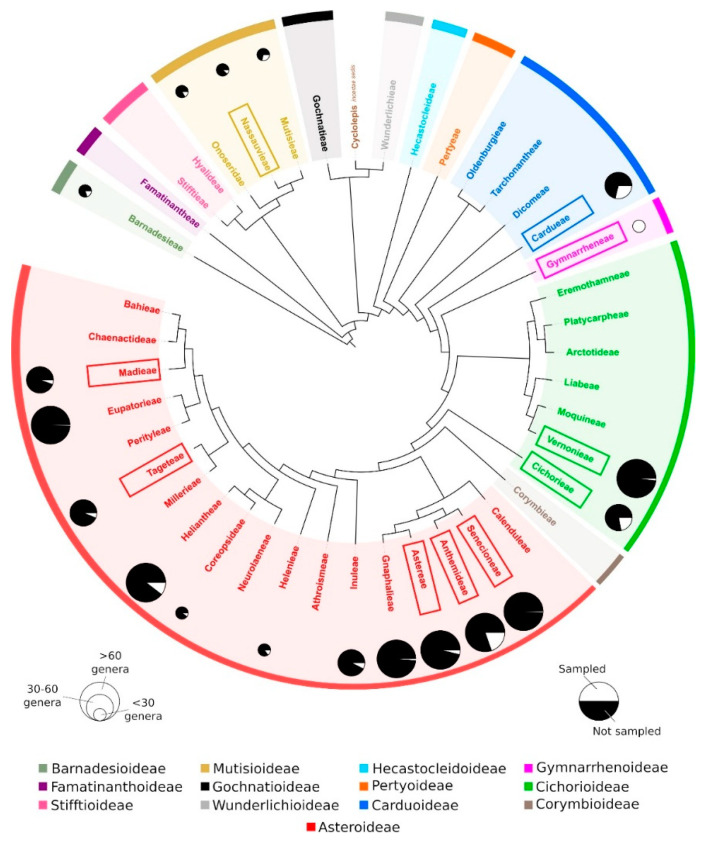
Overview of the phylogenetic relationships among tribes of Asteraceae [27]. The proportion of sampled genera for each analyzed tribe is indicated. Tribes showing ITR sites are boxed.

Altogether 176 species and three subspecies included in 115 genera have been analyzed for ITR. Of these, ITR signals have been detected in 36 species (20.11%) and 25 (21.74%) genera. The phylogenetic distribution of the species showing ITRs is shown in Figure 5 and Table 1.

No association between the presence of ITRs and the evolutionary placement of the species has been evidenced. ITRs have been recorded from 11 tribes, distributed across the phylogenetic tree of the family, including both early-diverging (Nassauvieae) and more recently diverged lineages (Cardueae, Gymnarrheneae, Vernonieae, Cichorieae, Senecioneae, Anthemidae, Astereae, Tageteae, and Madieae). 

**Figure 6:**
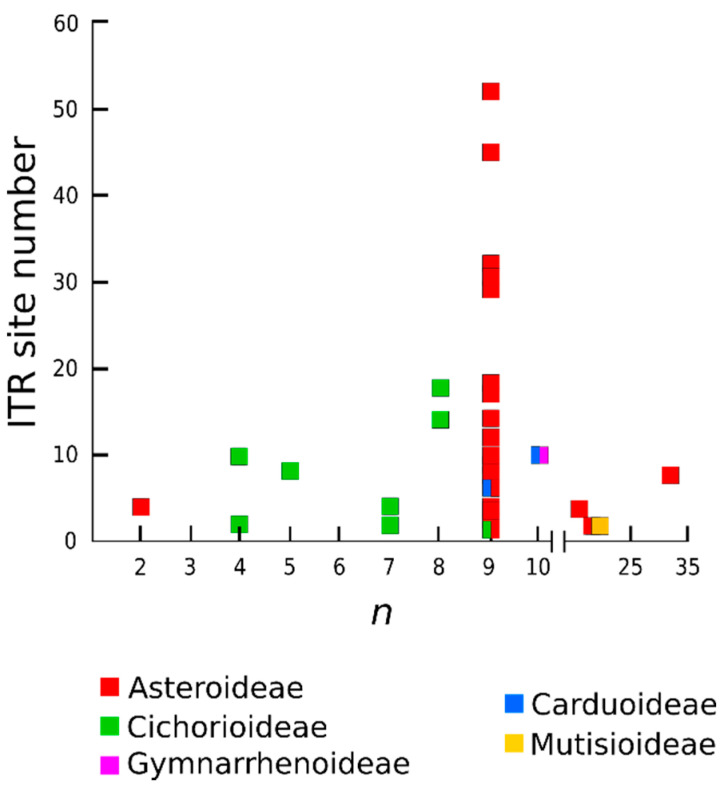
Relation between number of ITR sites and haploid chromosome number (*n*) in Asteraceae.

ITR sites are present across a wide range of chromosome numbers, from species with 2*n* = 4 (*Xanthisma gracile*) to 2*n* = 68 (*Porophyllum ruderale*). No apparent relation was detected between the number of ITR sites and the chromosome number of the species (Pearson correlation value r = −0.05, *p* = 0.6213; Figure 6), even if the results are analyzed separately for each subfamily (data not shown). This is clearly observed in Asteroideae, where species showing the same chromosome number (2*n* = 18) exhibit the widest range of ITR sites (2-52; Figure 6). 

**Figure 7:**
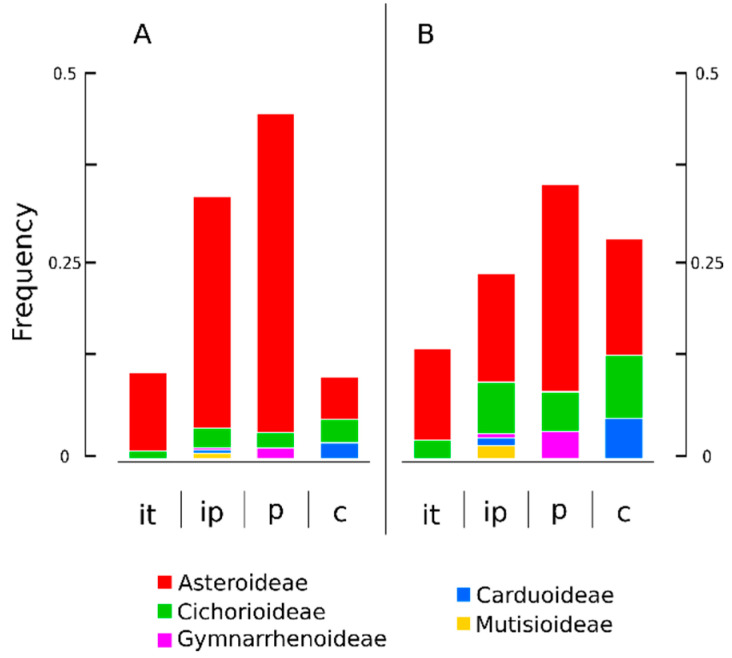
Longitudinal distribution of ITR sites along the chromosome arm in Asteraceae. The frequency in each chromosomal region is indicated. The chromosome arm was divided into four major domains of unequal size, i.e. centromeric (c), proximal (p), interstitial-proximal (ip), and interstitial-terminal (it). (**A**). Data from the whole dataset. (**B**) Data excluding *Anacyclus*.

ITR signals have been recorded from all regions of the chromosomes (centromeric, proximal and interstitial). However, the longitudinal distribution of ITR sites along the chromosome arms is uneven and their frequency was biased towards proximal and interstitial-proximal regions (Figure 7A). Since a substantial number of records were contributed from the large sampling available from *Anacyclus* [49], the data were also reanalysed without the values from this genus (Figure 7B).

In this case, there was an increase on the frequency of centromeric ITR sites and the concomitant decrease of the interstitial-proximal values. Centromeric signals were observed in species from *Gonospermum*, *Tanacetum*, *Achillea*, *Galactites* (Figure 2A), *Carlina* (Figure 2B), and *Hyoseris* (Figure 3C) (Table S1). However, non-centromeric ITR signals were also noted to co-occur for most of the samples. Only *Tanacetum parthenium* and *Galactites tomentosa* showed exclusively centromeric ITR signals (14 and 10, respectively).

## 3. Discussion

Asteraceae, with nearly 180 analyzed species and subspecies, is the best sampled family of seed plants for the presence of ITR sites in chromosomes [53]. In this paper, we have significantly increased the phylogenetic and taxonomic coverage previously known for this family [8,32,33,34,35,36,37,38,39,40,41,42,43,44,45,46,47,48,49,50]. Together with the data we provided in a previous micro-evolutionary level study [49], we are confident that an assessment of the phylogenetic signal, taxonomic utility, and evolutionary significance of ITR features in Asteraceae can be done. However, ITR data are still lacking for seven subfamilies (Corymbieae, Pertyeae, Hecastocleideae, Wunderlichieae, Gochnatieae, Stifftieae and Famatinantheae)—all representing small lineages containing a few species—and thus inferences made here have to be taken with some caution.

### 3.1. The Long Evolutionary History of Asteraceae May Have Erased Phylogenetic Signals of ITR Sites 

The perception that ITRs represent a labile chromosomal landmark in Asteraceae is evidenced in this study. Variation related to presence, site number, chromosomal location and copy number regarding the presence of ITRs is scattered across evolutionary lineages (Figure 5). No association at high (subfamily, tribe, subtribe) or low taxonomic levels (genus, species) can be supported with our data. This is illustrated in Anthemideae, the most thoroughly sampled tribe to date (23 genera, 56 species and one subspecies) where ITR variation among congeneric species and between closely related genera is outstanding. 

It should be emphasized that our survey was designed to prioritize the sampling of as many genera as possible at the expense of a low intrageneric coverage (ratio species/genus = 1.22). This precluded the assessment of ITR variation at low taxonomic ranks and shallow evolutionary levels (species and populations). Accordingly, it is likely that this biased sampling may have even underestimated the values of ITR variation at the generic and intraspecific levels. Our data obtained in *Sonchus tenerrimus*, where two intraspecific variants have yielded contrasting results concerning ITRs, point in this direction. Thus, it should not be discarded that analyzing more accessions from poorly sampled higher groups, where ITRs have not been detected, may reveal additional presences and even intra- and interspecific polymorphisms, as has been previously reported in *Anacyclus* [49] and *Tanacetum* [50]. However, we think that additional data would not contradict the present evidence that ITRs are not appropriate markers in tracing phylogenetic relationships and helping delimit taxonomic boundaries in Asteraceae. 

Whether the presence of ITR sites is an ancestral state in Asteraceae remains an open question deserving further studies. We have refrained from exploring evolutionary trends in ITR site change by maximum-likelihood or parsimony-based reconstruction due to the scarce data available for the early-diverging nodes. Available comparative data for related families indicate absence of ITRs (Table 1), including Calyceraceae, the sister family of Asteraceae. However, sampling is anecdotal and may be hardly used for outgroup purposes.

Another relevant question is whether ITR polymorphism is an odd genomic feature that is particularly represented in Asteraceae or it also affects other groups of angiosperms. In Fabaceae, the second most sampled family in flowering plants, the presence of ITRs have been assessed in 56 species included in 15 genera [7,8,44,54,55,56,57,58,59,60,61,62,63,64,65,66,67,68,69,70,71,72,73,74], where they were detected in seven not-closely related tribes (Cassiieae, Fabeae, Phaseoleae, Cicereae, Dalbergieae, Loteae and Trifolieae) [56,57,59,64,65,66,68,71,72,73,74]. Three genera belonging to different tribes showed both the presence and absence of ITRs (*Phaseolus*, *Senna*, *Vicia*) [8,44,57,61,65,67,70,71,73,74] and intraspecific variation has been reported in *Vicia faba* [8,44,61]. This pattern for ITR variation, with no evident phylogenetic signal nor taxonomic utility, is consistent with the available data in Asteraceae. 

It has been hypothesized that the origin of Asteraceae dates back to approximately 69.5 Ma in the late Cretaceous and subfamilies diverged between 64.75 and 43.25 Ma [75]. This long evolutionary history has been modulated by several episodes of genome duplication (see below), adaptive radiation, and Cretaceous–Tertiary extinctions that have triggered diversification rate shifts [29,75,76,77]. 

An accurate interpretation of karyotype changes can be compromised if chromosomal changes have occurred in ancient and complex evolutionary scenarios. The accumulation of old genomic changes (e.g., past genome duplications or paleopolyploidy, neopolyploidization, chromosome rearrangements leading to subsequent dysploidy and diploidization processes) in short periods of time can hinder elucidating the imprints left by each one of them in the evolutionary history of extant karyotypes [78]. In Asteraceae, hypotheses on chromosomal base number evolution were reported by [29]. In this work, hypothesized base numbers for each of the 36 main clades were superimposed on the backbone phylogeny provided by [79] and two main patterns of karyotype change emerged, (1) polyploidy is common in Asteraceae and occurs in most major clades and (2) descending dysploid events (in contrast to the rarer increasing dysploidy) are widespread across major lineages. Later on, [80], using explicit models of chromosome evolution, corroborated previous inferences and suggested that karyological evolution has been a very dynamic process in Asteraceae mainly shaped by polyploidy and descending dysploidy scenarios.

It can also be inferred from our previous work [49] that the deduced fast ITR turnover occurring at the population level in *Anacyclus* is not clearly associated to speciation events and it is likely that this finding also applies to other unrelated lineages. Thus, available evidence suggest that ITR features in Asteraceae do not convey a strong evolutionary signal to be used in deciphering phylogenetic relationships and are also of little, if any, value in helping to delimit taxonomic boundaries. On the contrary, patterns of hypervariability, such as those reported for *Anacyclus*, makes ITR a suitable marker for tracing genomic changes at micro-evolutionary scales using cytogenetic approaches. 

### 3.2. Are Centromeric ITR Signals Remnants of Dysploidy in Asteraceae?

In plants, the presence of ITR in centromeric and peri-centromeric locations has been considered to represent cytological landmarks related to dysploidy events e.g., [81,82,83,84,85,86]. There are two types of chromosomal rearrangements involved in dysploidy: end-to end chromosome fusions (symmetrical translocations) and fusion-fission cycle (Robertsonian rearrangements) between non-homologous chromosomes. Thus, on theoretical grounds ITR sites could be remnants of these karyological events. However, it has been pointed out that to acquire stability after chromosomal fusion events they should be accompanied by loss or inactivation of one of the centromeres and the resulting interstitial telomere sequences [78]. This view was modulated by [83], who indicated that the interstitial telomere sequences may be preserved during the chromosomal rearrangements because the telomere region of one chromosome is the breakpoint and therefore partially participates in the reciprocal translocation between the two acrocentric or telocentric chromosomes involved in the rearrangement. 

If ITR sites in Asteraceae were in fact cytogenetic signatures of disploidy, one question to be addressed is why ITR number contrasts between related species showing the same derived chromosome numbers. This is illustrated in the case of *Brachyschome dichromosomatica* and *Xanthisma gracile,* two species showing the highly reduced 2*n* = 4 chromosome number (the lowest chromosome number so far reported in seed plants). From the available evidence, it seems highly likely that the 2*n* = 4 karyotype was originated through many rounds of descendent disploidy events. Surprisingly, no ITRs have been documented in *B. dichromosomatica* [32], whereas in *X. gracile* two contrasting reports recording zero and four non-centromeric ITR sites are known [7,8,38]. 

Altogether, our data suggest that in Asteraceae the dynamics of ITR formation is too fast or labile to trace the complex karyological evolution that has been hypothesized to occur since the early diversification events in the family [75,80]. Further, part of such lability could be due to other types of karyological changes that erased previous ITR signs inconsistently across lineages.

## 4. Materials and Methods

### 4.1. Plant Materials

Seeds were collected in the field or were supplied by botanical gardens, plant breeding stations, research centres and commercial sources. The name and origin of the plant accessions used are provided in Table S2. Representative herbarium and seeds vouchers are deposited at the herbarium of the Botanical Garden of Valencia University (VAL) and the Germplasm banks of the Real Jardín Botánico de Madrid and the Botanical Garden of Valencia University. For comparative purposes, two species belonging to the related Calyceraceae and Goodeniaceae families (Asterales) were also studied.

### 4.2. FISH Analysis

Protocols for seed germination and the obtention of mitotic chromosomes follow the experimental procedures described in [87]. The telomeric sequence TTTAGGG was localised using the pAtT4 clone isolated from *Arabidopsis thaliana* [88]. The probe was labelled with biotin-16-dUTP through a nick translation procedure according to the manufacturer’s protocols (Roche, Germany). Probe detection was conducted using the method of [89] with the modifications described by [90].

### 4.3. Karyotype Analysis

For each accession, well spread metaphase plates were selected for assessing the chromosome number and the presence of ITRs. Individuals showing ITRs were further analyzed and the number of sites and their location along the chromosome were recorded. The chromosome arm was divided into four domains of equal size according to [91], i.e., centromeric (c), proximal (p), interstitial-proximal (ip), and interstitial-terminal (it) and the ITR sites detected were mapped on these chromosomal regions. Since most karyotypes of Asteraceae show metacentric chromosomes a reliable distinction between both arms was usually not feasible. Accordingly, the locations of ITR sites on both chromosome arms were pooled. Chromosome measurements were made on digital images using the computer application MicroMeasure version 3.2 [92].

The overview of the phylogenetic relationships among tribes is based on [27]. The tree was redrawn using the iTOL application [93] and Inkscape software. 

## Figures and Tables

**Table 1:** Taxonomic distribution of ITR sites in Asteraceae and some related families as assessed by FISH. For each major lineage (subfamily), the number of sampled tribes, genera and species is indicated; the number of taxa showing ITR sites in parenthesis. Data are from previous reports [8,32,33,34,35,36,37,38,39,40,41,42,43,44,45,46,47,48,49,50] and our own results.

Family	Subfamily	No. of Tribes	No. of Genera	No. of Species	No. of Subsp.
Asteraceae	Asteroideae	11 (2)	62 (15)	97 (24)	1
	Carduoideae	1 (1)	21 (2)	31 (2)	1
	Cichorioideae	2 (2)	21 (6)	35 (8)	1
	Gymnarrhenoideae	1 (1)	1 (1)	1 (1)	0
	Mutisioideae	3 (1)	8 (1)	10 (1)	0
	Barnadesioideae	1 (0)	2 (0)	2 (0)	0
Calyceraceae			1 (0)	1(0)	0
Goodeniaceae			2 (0)	2(0)	0
Campanulaceae			1 (0)	1 (0)	0
Stylidaceae			1 (0)	1 (0)	0

## Data Availability

Not applicable.

## Supplementary Materials

The following are available online at https://www.mdpi.com/article/10.3390/plants10122794/s1, Table S1: Distribution of ITR sites in Asteraceae and related families of Asterales, Table S2: List of studied accessions and sources of analyzed material by FISH.
