# Supplementary material for: Interstitial Arabidopsis-Type Telomeric Repeats in Asteraceae
Source: Plants (Basel). 2021 Dec 17;10(12):2794. doi: 10.3390/plants10122794 (PMC8705333; doi:10.3390/plants10122794)
Supplement: Supplementary file 1 [file plants-10-02794-s001.zip › Table S1.pdf]

Table S1. Distribution of ITR sites in Asteraceae and related families of Asterales. E: occurrence of extra chromosomes. +: presence. nr: not reported. [1] Each chromosome arm was divided into four regions according to Roa and Guerra (2012): centromeric (c), proximal (p), interstitial-proximal (ip), interstitial-terminal (it). [2] 1: This work; 2: Fuchs et al. (1995); 3: Houben et al. (1999); 4: Adams et al. (2001); 5: Borgen et al. (2003); 6: Pires et al. (2004); 7: Abd El-Twab and Kondo (2006); 8: Abd El-Twab and Kondo (2007); 9: Hanmoto et al. (2007); 10: Matoba et al. (2007); 11: Dydak et al. (2009); 12: García et al. (2009); 13: Matoba and Uchiyama (2009); 14: Shibata and Hizume (2011); 15: Li et al. (2012); 16: Cuyacot et al. (2016); 17: Cuyacot et al. (2017); 18: Mancia et al. (2017); 19: Rosato et al. (2018); 20: Mlinarec et al. (2019); 21: Abd El-Twab et al. (2009).

	Subfamily	Tribe	Subtribe	2n	ITR site number	Location[1]	References[2]
**Asteraceae**							
Anthemis arvensis L.	Asteroideae	Anthemideae	Anthemidinae	18	0	-	1
Anthemis cotula L.				18	2	it	1
Anthemis maritima L.				36	0	-	1
Anthemis rosularis P. Fraga & Rosselló				18	0	-	1
Anthemis tinctoria L.				18	3	p, ip	1
Cota nigellifolia (Boiss.) Alv. Fern. & Vitales				18	52	p, ip, it	1
Gonospermum fruticosum (Buch) Less.				18	29	c, p, ip, it	1
Tanacetum cinerariifolium (Trevir.) Sch. Bip.				18	32	c, p, ip, it	20

Tanacetum parthenium (L.) Sch. Bip.		18	14, 0	c	1; 4
Tanacetum ptarmiciflorum Sch. Bip.		18	+	nr	5
Tanacetum vulgare L.		18	2, 0	ip	1; 4
Artemisia bigelovii A. Gray	Artemisiinae	18, 36	0	-	12
Artemisia feddei H. Lév. & Vanio		16	0	-	13
Artemisia keiskeana Miq.		18	0	-	13
Artemisia nova A. Nelson		18, 36	0	-	12
Artemisia princeps Pamp.		34	0	-	13
Artemisia tridentata Nutt. subsp. *tridentata*		18, 36	0	-	12
Artemisia tripartita Rybd. subsp. *tripartita*		18, 36	0	-	12
Artemisia vulgaris L.		16	0	-	1
Crossostephium artemisioides Less.		18	0	-	14
Chrysanthemum lavandulifolium (Fisch. ex Trautv.) Makino		18	0	-	16
Chrysanthemum zawadskii Herbich		36, 54	0	-	7; 17
Leucanthemella linearis (Matsum. ex Matsum.) Tzvelev		18	12	i	8
Leucanthemella serotina (L.) Tzvelev		18	0	-	1
Nipponanthemum nipponicum (Franch. ex Maxim.) Kitam.		18	0	-	8; 14
Lasiospermum bipinnatum (Thunb.) Druce	Athanasiinae	18+2E	0	-	1

Cotula socialis Hilliard	Cotulinae	18	0	-	1
Argyranthemum broussonetii (Pers.) Humphries	Glebionidinae	18	0	-	5
Argyranthemum haouarytheum Humphries & Bramwell		18	0	-	1
Argyranthemum frutescens (L.) Sch. Bip.		18	0	-	5
Argyranthemum lemsii Humphries		18	0	-	5
Argyranthemum sundingii L. Borgen		18	0	-	5
Glebionis coronaria (L.) Cass. ex Spach		18	0	-	1; 14
Glebionis segetum (L.) Fourr.		18	+	nr	15
Leucanthemum graminifolium (L.) Lam.	Leucanthemiinae	ca. 36	2	ip	1
Mauranthemum ebusitanum (Vogt) N. Torres & Rosselló		18	0	-	1
Rhodanthemum arundanum (Boiss.) B. H. Wilcox, K. Bremer & Humphries		18	0	-	1
Achillea distans Waldst. & Kit. ex Willd.	Matricariinae	54	0	-	1
Achillea ligustica All.		18+1E	17	c, p	1
Achillea maritima (L.) Ehrend. & Y. P. Guo		18	0	-	1
Achillea millefolium L.		54	0	-	21
Anacyclus atlanticus Litard. & Maire		18	6, 8, 17	p, ip, it	19
Anacyclus clavatus (Desf.) Pers.		18	0, 2, 3, 4, 6, 8, 9, 12, 14	p, ip	19
Anacyclus homogamos (Maire) Humphries		18	0	-	19

Anacyclus linearilobus Boiss. & Reuter			18	19	p, ip	19
Anacyclus maroccanus (Ball) Ball			18	0	-	19
Anacyclus monanthos (L.) Thell.			18	0, 4	p	19
Anacyclus pyrethrum (L.) Link			18	26, 30, 45	p, ip, it	19
Anacyclus radiatus Loisel. subsp. *coronatus* (Murb.) Humphries			18	0	-	19
Anacyclus radiatus Loisel. subsp. *radiatus* (Murb.) Humphries			18	0	-	19
Anacyclus valentinus L.			18	0, 2, 3, 4, 5, 6, 10	p, ip, it	19
Matricaria chamomilla L.			18	+	nr	14
Chamaemelum nobile (L.) All.		Santolininae	18	0	-	1
Cladanthus arabicus (L.) Cass.			18	0	-	1
Cladanthus scariosus (Ball) Oberpr. & Vogt			18	12	p, ip, it	1
Ursinia speciosa DC.		Ursiinae	16	0	-	1
Lonas annua (L.) Vines & Druce		incertae sedis	18	0	-	1
Senecio vulgaris L.	Senecioneae		40	2	p	1
Brachyscome dichromosomatica C. R. Carter	Astereae		4	0	-	3
Erigeron acer L.			18	0	-	1
Erigeron karvinskianus DC.			36	0	-	1

Heterotheca villosa (Pursh) Shinners			18	0	-	1
Symphyotrichium nova-belgii (L.) G. L. Nesom			48	0	-	1
Xanthisma gracile (Nutt.) D. R. Morgan & R. L. Hartm.			4	4, 0	it	2; 9
Xanthisma texanum DC.			8	0	-	1
Bellium crassifolium Moris		Bellidinae	18	0	-	1
Noticastrum diffusum (Pers.) Cabrera		Chrysopsidinae	18	0	-	1
Felicia tenella (L.) Nees		Homochrominae	18	0	-	1
Grindelia chiloensis (Cornel.) Cabrera		Machaerantherinae	12	0	-	1
Coreopsis gigantea (Kellog) H. M. Hall	Coreopsideae		24	0	-	1
Cosmos bipinnatus Cav.			24	0	-	1
Dahlia merckii Lehm.			36	0	-	1
Liatris spicata (L.) Willd.	Eupatorieae		20	0	-	1
Anaphalis margaritacea (L.) Benth. & Hook. f.	Gnaphalieae		28	0	-	1
Pycnosorus globosus F. L. Bauer ex Benth.			20	0	-	1
Helenium aromaticum (Hook.) L. H. Bailey	Helenieae	Gaillardiinae	34	0	-	1
Hymenoxys hoopesii (A.Gray) Bierner		Tetraneurinae	30	0	-	1

Echinacea purpurea (L.) Moench	Heliantheae		22	0	-	1
Helianthus annuus L.			34	0	-	2
Iva xanthiifolia Nutt.			36	0	-	1
Lindheimera texana A. Gray & Engelm.			16	0	-	1
Silphium perfoliatum L.			14	0	-	1
Synedrella nodiflora (L.) Gaertn.			36	0	-	1
Tithonia rotundifolia (Mill.) S. F. Blake			34	0	-	1
Xanthium italicum Moretti			36	0	-	1
Schkuhria pinnata (Lam.) Kuntze ex Thell.		Bahieae	20	0	-	1
Guizotia abyssinica (L. f.) Cass.		Millerieae	30	0	-	1
Melampodium sp.			24	0	-	1
Tridax trilobata (Cav.) Hemsl.			20	0	-	1
Chiliadenus glutinosus (L.) Fourr.	Inuleae	Inulinae	16	0	-	1
Dittrichia viscosa (L.) Greuter			18	0	-	1
Inula helenium L.			20	0	-	1
Pallenis spinosa (L.) Cass.			10	0	-	1
Perralderia paui Font Quer		Plucheinae	18	0	-	1
Arnica sachalinensis (Regel) A. Gray	Madieae	Arnicinae	38	0	-	1

Madia sativa Molina			Madiinae	32	4	it	1
Flaveria trinervia (Spreng.) C. Mohr		Tageteae		36	0	-	1
Porophyllum ruderale (Jacq.) Cass.				68	8	p, ip	1
Barnadesia spinosa L.	Barnadesioideae	Barnadesieae		50	0	-	1
Chuquiraga jussieui J.F. Gmel.				ca. 54	0	-	1
Arctium lappa L.	Carduoideae	Cardueae	Carduinae	36	0	-	1
Carduus nigrescens Vill. subsp. *assoi* Willk.				20+3E	0	-	1
Cirsium vulgare (Savi) Ten.				68	0	-	1
Cynara scolymus L.				34	0	-	1
Galactites tomentosa Moench				20	10	c	1
Jurinea ledebourii Bunge				36	0	-	1
Onopordum acaulon L.				34	0	-	1
Onopordum tauricum Willd.				34	0	-	1
Ptilostemon gnaphaloides Soják				32	0	-	1
Silybum marianum (L.) Gaertn.				34	0	-	1
Staehelina dubia L.				30	0	-	1
Xeranthemum annuum L.				12	0	-	1

Carlina hispanica Lam.	Carlininae	18	6	c, ip	1
Carduncellus monspelliensium All.	Centaureinae	48	0	-	1
Carthamus balearicus (J.J. Rodr.) Greuter		24	0	-	1
Carthamus tinctorius L.		24	0	-	1; 18
Centaurea jacea L.		44	0	-	11
Centaurea montana L.		44	0	-	1
Centaurea nigra L.		22	0	-	1
Centaurea phrygia L.		22	0	-	11
Centaurea oxylepis (Wimm. & Grab.) Hayek		44	0	-	11
Centaurea seridis L.		33	0	-	1
Cheirolophus canariensis (Brouss. ex Willd.) Holub subsp. *canariensis*		30	0	-	1
Cheirolophus canariensis subsp. *subexpinnata* (Burch.) G. Kunkel		30	0	-	1
Cheirolophus webbianus (Sch. Bip.) Holub		30	0	-	1
Klasea nudicaulis (L.) Fourr.		30	0	-	1
Klasea pinnatifida (Cav.) Talavera		30	0	-	1
Lamottea diania (Webb) G. López		24	0	-	1
Mantisalca salmantica (L.) Briq. & Cavill.		22	0	-	1

Rhaponticum coniferum (L.) Greuter				26	0	-	1
Echinops ritro L.			Echinopsinae	32	0	-	1
Echinops sphaerocephalus L.				30	0	-	1
Arctotis venusta Norl.	Cichorioideae	Cichorieae	Arctotidinae	18	0	-	1
Arctotheca calendula (L.) Levyns				18	0	-	1
Dymondia margaretae Compton				18	2	p	1
Cichorium intybus L.			Cichoriinae	18	0	-	1
Tolpis barbata (L.) Gaertn.				18	0	-	1
Crepis capillaris (L.) Wallr.			Crepidinae	6	0	-	2
Crepis triasii (Cambess.) Fr.				8	0	-	1
Taraxacum sp.				24	0	-	1
Andryala integrifolia L.			Hieraciinae	18	0	-	1
Hyoseris radiata L.			Hyoseridinae	16	14	c, p, ip	1
Hyoseris taurina (Pamp.) Martinoli				16	18	c, p	1
Reichardia picroides (L.) Roth				14	0	-	1
Sonchus masguindalii Pau & Font Quer				18	0	-	1
Sonchus tenerrimus L. subsp. *dianae* (Lacaita ex Willk.) Malag.				14	0	-	1

Sonchus tenerrimus L. subsp. *tenerrimus*		14	2	ip	1
Helminthotheca echioides (L.) Holub	Hypochaeridinae	10	8	ip	1
Leontodon hispidus L.		14	0	-	2
Leontodon longirostris (Finch & P. D. Sell) Talavera		8	10	ip,it	1
Leontodon tuberosus L.		8+2E	2	p	1
Urospermum dalechampii (L.) Scop. ex F. W. Schmidt		14	0	-	1
Urospermum picroides (L.) Scop. ex F. W. Schmidt		10	0	-	1
Lactuca saligna L.	Lactuceae	18	0	-	10
Lactuca sativa L.		18	0	-	10
Lactuca serriola L.		18	0	-	1; 10
Lactuca virosa Habl.		18	0	-	10
Launaea arborescens (Batt.) Murb.		14	0	-	1
Launaea cervicornis (Boiss.) Font Quer & Rothm.		18	0	-	1
Catananche caerulea L.	Scolyminae	18	0	-	1
Scorzonera laciniata Jacq.	Scorzonerinae	14	0	-	1
Tragopogon dubius Scop.		12	0	-	1; 6

Tragopogon miscellus Ownbey				24	0	-	6
Tragopogon mirus Ownbey				24	0	-	6
Tragopogon pratensis L.				12	0	-	6
Tragopogon porrifolius L.				12	0	-	6
Stokesia laevis (Hill) Greene		Vernonieae	Stokesiinae	14	4	ip, it	1
Vernonia fasciculata Michx.			Vernoniinae	34	0	-	1
Gymnarrhena micrantha Desf.	Gymnarhenoideae	Gymnarheneae		20	10	p, ip	1
Chaetanthera microphylla (Cass.) Hook. & Arn.	Mutisioideae	Mutisieae		24	0	-	1
Chaptalia arechavaletae Arechav.				ca. 100	0	-	1
Chaptalia exscapa (Pers.) Baker				100	0	-	1
Leibnitzia anandria (L.) Turcz.				46	0	-	1
Mutisia coccinea A. St.-Hil.				52	0	-	1
Nassauvia serpens d'Urv.		Naussavieae		22	0	-	1
Nassauvia sprengelioides DC.				44	4	ip	1
Proustia pyrifolia DC.				52	0	-	1
Trixis californica Kellogg				54	0	-	1
Onoseris alata Rusby		Onoserideae		ca. 46	0	-	1

**Calyceraceae**				
Calycera herbacea Cav.	42	0	-	1
**Campanulaceae**				
Lobelia erinus L.	28	0	-	14
**Goodeniaceae**				
Goodenia fascicularis F. Muell. & Tate	16	0	-	1
Leschenaultia formosa R. Br.	nr	0	-	14
**Stylidaceae**				
Candollea debilis F.Muell	nr	0	-	14
