# Supplementary material for: Interstitial Arabidopsis-Type Telomeric Repeats in Asteraceae
Source: Plants (Basel). 2021 Dec 17;10(12):2794. doi: 10.3390/plants10122794 (PMC8705333; doi:10.3390/plants10122794)
Supplement: Supplementary file 1 [file plants-10-02794-s001.zip › Table S2.pdf]

Table S2. List of studied accessions and sources of analysed material by FISH. Seeds from wild origins accessed from personal recollections and research institutions are indicated [Inés Alvaréz (IA), Vicente Colomer (VC), Leopoldo de Simone (LdS), Elena Estrelles (EE), Pere Fraga (PF), Ana Ibars (AI), Javier Fabado (JF), Antonio Galán (AGM), Alexis J. Maravilla (AJMA), Gonzalo Nieto Feliner (GNF), Josefa Prieto (JP), Jesús Riera (JR), Marcela Rosato (MR), Josep A. Rosselló (JAR)] and research institutions [Botanischer Garten Bielefeld (BEILF); Botanischer Garten Berlin-Dahlem (BGB-D); Botanischer Garten der Johannes Gutenberg-Universität Mainz (MJG); Botanischer Garten der Universität Leipzig (LZ); Botanischer Garten der Universität Potsdam (POTSD); Botanical Garden of Teplice (TEBLI); Centro para la Investigación y Experimentación Forestal (CIEF); Civico Orto Botanico di Triste (TSM); Conservatoire et Jardin Botaniques Gèneve (CJB); Denver Botanic Gardens (KHD), Jardí Botànic de Barcelona (BC); Jardín Botánico Canario Viera y Clavijo (JBCVC); Jardin Botanique de Clermont-Ferrand (CLER); Jardí Botànic Marimurtra (BLAN); Jardí Botànic de la Universitat de València (VAL); Millennium Seed Bank (MSB); Real Jardín Botánico de Madrid (RJB-CSIC); Research Institute of Forests and Rangelands (RIFR); Siberian Botanical Garden Tomsk State University (TK); Universitatea de Medicină și Farmacie din Târgu Mureș] (UMFST).

Taxon	Origin	Source (accession number)	Voucher
Achillea distans Waldst. & Kit. ex Willd.	RO-0-B-0380603. Romania, Hunedoara, P National Retezat (Bot. Garten Iasi)	BGB-D	BRL 530
A. ligustica All.	IT-0-B-2530203. Italy, Toscana, Monti Pisani, Santallago	BGB-D	BRL 531
A. maritima (L.) Ehrend. & Y.P.Guo	Spain, Valencia, Saler. *AJMA*	VAL (247532)	
Anaphalis margaritacea (L.) Benth. & Hook.f.	XX-0-LZ-AN-47-2010	LZ	JBVAL-CE 2798
Andryala integrifolia L.	Spain, Valencia, Algemesí. *AJMA*	VAL	JBVAL-CE C2862
Anthemis arvensis L.	XX-0-LZ-SYS-26-2011	LZ	JBVAL-CE C2799
A. cotula L.	GR-0-B-2403992. Greece, Makedonien, Nomos	BGB-D	BRL 551
A. maritima L.	Spain, Balearic Islands, Menorca. *PF*	VAL	JBVAL-CE C2863
A. rosularis P.Fraga & Rosselló	Spain, Balearic Islands, Menorca. *PF*	VAL	JBVAL-CE C2864
A. tinctoria L.	unknown	VAL (C 25)	JBVAL-CE C2865
Arctium lappa L.	CH0G-20170054	CJB (1060)	JBVAL-CE 2730
Arctotheca calendula (L.) Levyns	XX-0-MJG-199704110	MJG (120)	JBVAL-CE C2866
Arctotis venusta Norl.	unknown	TEBLI (without number)	JBVAL-CE C2867
Argyranthemum haouarytheum Humphries & Bramwell	Spain, Canary Islands, Gran Canaria	JBCVC (PFN33)	JBVAL-CE C2868
Arnica sachalinensis (Regel) A.Gray	unknown	BEILF (55)	JBVAL-CE 2778
Artemisia vulgaris L.	unknown	VAL (4419-90)	JBVAL-CE C2869

Barnadesia spinosa L.	Ecuador, Quito, cultivated at Museum of Pumapungo. *GNF*	RJB-CSIC	4981GN
Bellium crassifolium Moris	unknown	VAL (44-99)	JBVAL-CE C2872
Calycera herbacea Cav.	Chile	Commercial accession	JBVAL-CE C2969
Carduncellus monspeliensis St.-Lag	Spain, Valencia, Alzira, La Casella. *AJMA*	VAL (241078)	JBVAL-CE C2873
Carduus nigrescens subsp. *assoi* Willk.	Spain, Valencia, Teresa de Cofrentes, Cuesta Vallart. *MR & VC*	VAL (241075)	JBVAL-CE C2874
Carlina hispanica Lam.	Spain, Valencia, Jarafuel, Barranco del Agua. *MR & VC*	VAL	JBVAL-CE-C2995
Carthamus balearicus (J.J. Rodr.) Greuter	Spain, Balearic Islands, Menorca	VAL (27-97)	JBVAL-CE-C2997
C. tinctorius L.	unknown	TSM (without number)	JBVAL-CE C2875
Catananche caerulea L.	Spain, Valencia, Alzira, PNM La Murta. *AJMA*	VAL (241094)	JBVAL-CE C2876
Centaurea montana L.	unknown	RJB-CSIC	ES-0-MA-00409
C. nigra L.	unknown	RJB-CSIC	ES-0-MA-00410
C. seridis L.	unknown	VAL (2146-90)	JBVAL-CE C2877
Chaetanthera microphylla (Cass.) Hook. & Arn.	Chile	Commercial accession	JBVAL-CE C2951
Chamaemelum nobile (L.) All.	unknown	CLER (2709)	JBVAL-CE C2878
Chaptalia arechavaletae Arechav.	XX-0-B-0633174	BGB-D (482, 541)	JBVAL-CE C2879
Chaptalia exscapa (Pers.) Baker	Chile	Comercial accession	JBVAL-CE C2952
Cheirolophus canariensis (Brouss. ex Willd.) Holub	Spain, Canary Islands, Tenerife, Teno- ES-0-B-0840385	BGB-D (482, 542)	JBVAL-CE-C2987
C. canariensis subsp. *subexpinnatus* (Burchard) G. Kunkel	Spain, Barcelona, JBB. N12503	BC (1712)	JBVAL-CE-C2986
C. webbianus (Sch.Bip.) Holub	Spain, Barcelona, JBB. N12481	BC (1714)	JBVAL-CE-C2988
Chiliadenus glutinosus (L.) Fourr.	Spain, Valencia, Lliria, Cotó del Català	CIEF (V662)	JBVAL-CE C2904
Chuquiraga jussieui J.F. Gmel.	Ecuador, Quito, Cruz Loma, Rucu Pinchincha route. *GNF*	RJB-CSIC	4982GN
Cichorium intybus L.	Spain, Valencia, Algemesí. *AJMA*	VAL	JBVAL-CE C2880
Cirsium vulgare (Savi) Ten.	Spain, Valencia, Algemesí. *AJMA*	VAL	JBVAL-CE C2881
Cladanthus arabicus (L.) Cass.	unknown	CLER (2715)	JBVAL-CE C2882
C. scariosus (Ball) Oberpr. & Vogt	Morocco, Imlil, Toubkal National Park	RJB-CSIC	IA 2352
Coreopsis gigantea (Kellog) H.M. Hall	unknown	VAL (244b)	JBVAL-CE C2884
Cosmos bipinnatus Cav.	unknown	RJB-CSIC	ES-0-MA-00501
Cota nigellifolia (Boiss.) Alv. Fern. & Vitales	Iran, Kermanshah, Bisoton Mozaffarian	RIFR	TARI 83549
Cotula socialis Hilliard	unknown	TEBLI	JBVAL-CE C2883
Crepis triasii (Cambess.) Fr.	Spain, Balearic Islands, Mallorca	VAL (328-9)	JBVAL-CE C2885
Cynara scolymus L.	unknown	RJB-CSIC	ES-0-MA-00547
Dahlia merckii Lehm.	XX-0-MJG-199709202	MJG (150)	JBVAL-CE C2886

Dittrichia viscosa (L.) Greuter	Spain, Valencia, Simat de la Valldigna, Font del Cirer	CIEF (V1052)	JBVAL-CE C2887
Dymondia margaretae Compton	unknown	VAL (241091)	JBVAL-CE C2888
Echinacea purpurea (L.) Moench	unknown	TK (58)	JBVAL-CE C2889
Echinops ritro L.	Spain, Valencia, Bicorp, Rio Fraile. *MR & VC*	VAL (241076)	JBVAL-CE C2890
E. sphaerocephalus L.	Spain, Lleida, Cornellana. N13461	BC (1860)	JBVAL-CE C2891
Erigeron acer L.	CH0G-20171874	CJB (1081)	JBVAL-CE 2733
E. karvinskianus DC.	unknown	VAL (241087)	JBVAL-CE C2892
Felicia tenella (L.) Nees	XX-0-HAL-2889 2013-0478	POTSD (0565)	JBVAL-CE 2757
Flaveria trinervia (Spreng.) C. Mohr	XX-0-MJG-199709209	MJG (155)	JBVAL-CE C2893
Galactites tomentosa Moench	Spain, Valencia, Alzira. *AJMA*	VAL	JBVAL-CE C2894
Glebionis coronaria (L.) Cass. ex Spach	Spain, Valencia, La Pobla de Vallbona, Parc Cap de l´horta. *MR & VC*	VAL (241081)	JBVAL-CE C2895
Gonospermum fruticosum (Buch) Less.	Spain, Canary Islands, Tenerife, Costa de Acentejo, La Victoria	JBCVC (4675/B)	JBVAL-CE C2896
Goodenia fascicularis F. Muell. & Tate	unknown	MSB (0086860)	JBVAL-CE C2897
Grindelia chiloensis (Cornel.) Cabrera	Argentina	UMFST (114)	JBVAL-CE 2836
Gymnarrhena micrantha Desf.	unknown	MSB (0416278)	JBVAL-CE C2898
Guizotia abyssinica (L.f.) Cass.	XX-0-HOH-SYS-13367	LZ	JBVAL-CE 2803
Helenium aromaticum (Hook.) L.H.Bailey	unknown	VAL (241089)	JBVAL-CE C2899
Helminthotheca echioides (L.) Holub.	unknown	VAL	JBVAL-CE C2922
Heterotheca villosa (Pursh) Shinners	Canada, British Columbia, Nelson, Cranbrook. CA-0-B-2008004	BGB-D (482, 620)	JBVAL-CE-C2989
Hymenoxys hoopesii (A.Gray) Bierner	unknown	KHD (61)	JBVAL-CE C2901
Hyoseris radiata L.	XX-0-MJG-200307020	MJG (167)	JBVAL-CE C2902
H. taurina (Pamp.) Martinoli	Italy, Sicilia, Capo Gallo. *LdS*	VAL	JBVAL-CE C2903
Inula helenium L.	unknown	RJB-CSIC	ES-0-MA-00777
Iva xanthiifolia Nutt.	XX-0-LZ-AD-261-2006	LZ	JBVAL-CE 2804
Jurinea ledebourii Bunge	Nursery of JBB. N16972	BC (3918)	JBVAL-CE C2905
Klasea nudicaulis (L.) Fourr.	Spain, Teruel, Valdeconejos. N15801	BC (2358)	JBVAL-CE C2931
K. pinnatifida (Cav.) Talavera	Spain, Valencia, Valencia, Alzira. *AJMA*	VAL (241096)	JBVAL-CE C2906
Lactuca serriola L.	Spain, Valencia, Algemesí. *AJMA*	VAL	JBVAL-CE C2907
Lamottea diania (Webb) G. López	unknown	VAL (241093)	JBVAL-CE C2908
Lasiospermum bipinnatum (Thunb.) Druce	XX-0-MJG-199707712	MJG (174)	JBVAL-CE C2909
Launaea arborescens (Batt.) Murb.	Spain, Almeria. *MR*	VAL	JBVAL-CE C2910

Launaea cervicornis (Boiss.) Font Quer & Rothm.	unknown	VAL (07-2016	JBVAL-CE-C2990
Leibnitzia anandria (L.) Turcz.	XX-0-POTSD-1999-0158	POTSD (0578)	JBVAL-CE 2759
Leontodon longirostris (Finch & P.D. Sell) Talavera	Spain, Benifato, Serra d'Aitana, Partagat. *JR* & *JF*	VAL	JBVAL-CE C2911
L. tuberosus L.	unknown	VAL (241085, 4258-90)	JBVAL-CE C2912
Leucanthemella serotina (L.) Tzvelev	unknown	BEILF (189)	JBVAL-CE 2784
Leucanthemum graminifolium (L.) Lam.	unknown	RJB-CSIC	ES-0-MA-00828
Liatris spicata (L.) Willd.	unknown	BEILF (193)	JBVAL-CE 2786
Lindheimera texana A. Gray & Engelm.	XX-0-MJG-19--46780	MJG (179)	JBVAL-CE C2913
Lonas annua (L.) Vines & Druce	XX-0-MJG-19--46790	MJG (180)	JBVAL-CE C2914
Madia sativa Molina	XX-0-MJG-19--62730	TEBLI (77)	JBVAL-CE C2915
Mantisalca salmantica (L.) Briq. & Cavill.	Spain, Valencia, Alzira, PNM La Murta. *AJMA*	VAL (241095)	JBVAL-CE C2916,
Mauranthemum ebusitanum (Vogt) N. Torres & Rosselló	Spain, Balearic Islands, Eivissa, Puig d´en Serra. *JAR*	VAL	JBVAL-CE C2917
Melampodium sp.	XX-0-MJG-19--46810	MJG (184)	JBVAL-CE C2918
Mutisia coccinea A.St.-Hil.	unknown	BLAN (without number)	JBVAL-CE C2919
Nassauvia serpens d'Urv.	unknown	MSB (0451923)	JBVAL-CE C2920
N. sprengelioides DC.	Chile	Commercial accession	JBVAL-CE C2964
Noticastrum diffusum (Pers.) Cabrera	Argentina, Buenos Aires, S. Tandil, S. del Tigre. AR-0-B-1210497	BGB-D (482, 686)	JBVAL-CE-C2991
Onopordum acaulon L.	Spain, Valencia, Teresa de Cofrentes, Cuesta Vallart. *MR & VC*	VAL	JBVAL-CE C2921
O. tauricum Willd.	unknown	RJB-CSIC	ES-0-MA-00986
Onoseris alata Rusby	McAlpin s/n; cultivated in Las Cruces Research Station and Wilson Botanical Garden (Costa Rica)	Jeff Barter 59-883 (Hawaii, USA)	JBVAL-CE-C2992
Pallenis spinosa (L.) Cass.	Spain, Valencia, Jarafuel, Cañadilla. *MR & VC*	VAL	JBVAL-CE C2871
Perralderia paui Font Quer	Morocco, Fés-Meknés, Taza, Tizi-Ouazli. MA-0-B-0990417	BGB-D (482, 701)	JBVAL-CE-C2993
Porophyllum ruderale (Jacq.) Cass.	XX-0-TEBLI-01292	TEBLI (79)	JBVAL-CE C2923
Proustia pyrifolia DC.	Chile	Commercial accession	JBVAL-CE C2968
Ptilostemon gnaphaloides Soják	unknown	VAL (241092, 209-99)	JBVAL-CE C2924
Pycnosorus globosus F.L. Bauer ex Benth.	unknown	VAL (247-14)	JBVAL-CE C2925
Reichardia picroides (L.) Roth	Spain, Xàbia, Playa de la Barraca. *AI, JP, EE*	VAL	JBVAL-BG-62B2017
Rhaponticum coniferum (L.) Greuter	Spain, Valencia, Jarafuel, Campillo. *MR & VC*	VAL	JBVAL-CE C2926
Rhodanthemum arundanum (Boiss.) B.H. Wilcox, K. Bremer & Humphries	Morocco, Fés-Meknès, Boulmane, Outat El Haj. MA-0-B-0990717	BGB-D (482, 737)	JBVAL-CE C2927
Schkuhria pinnata (Lam.) Kuntze ex Thell.	XX-0-MJG-200403211	MJG (198)	JBVAL-CE C2928

Scorzonera laciniata Jacq.	Spain, Alicante, Villena. *AJMA*	VAL	JBVAL-CE C2929
Senecio vulgaris L.	Spain, Valencia, Algemesí. *AJMA*	VAL	JBVAL-CE C2930
Silphium perfoliatum L.	unknown	VAL (241083, 1204-90)	JBVAL-CE C2932
Silybum marianum (L.) Gaertn.	unknown	VAL (470-15)	JBVAL-CE C2933
Sonchus masguindalii Pau & Font Quer	Spain, Canary Islands, Tenerife, Anaga-Gebirge. ES-0-B-0411200	BGB-D (482, 786)	JBVAL-CE-C2996
S. tenerrimus L. subsp. *tenerrimus*	Spain, Murcia, Lorca. *AJMA*	VAL	VAL-242610
S. tenerrimus subsp. *dianae* Malag.	Spain, Alicante, Xàbia. *AJMA & MR*	VAL	JBVAL-CE-C2994
Staehelina dubia L.	Spain, Tarragona, Mas de la Franqueta. N13264	BC (1752)	JBVAL-CE C2934
Stokesia laevis (Hill) Greene	XX-0-MJG-19--46970	MJG (206)	JBVAL-CE C2935
Symphyotrichum novi-belgii (L.) G.L. Nesom.	unknown	VAL (74-97)	JBVAL-CE C2870
Synedrella nodiflora (L.) Gaertn.	XX-0-MJG-199703910	MJG (208)	JBVAL-CE C2936
Tanacetum parthenium (L.) Sch. Bip.	unknown	BEILF (320)	JBVAL-CE 2789
T. vulgare L.	Humboldt-Univ. Berlin. DE-0-B-0201195	BGB-D	BRL 891
Taraxacum sp.	Spain, Zamora, Fuentesauco. *AGM*	VAL (247533)	
Tithonia rotundifolia (Mill.) S.F. Blake	XX-0-MJG-19--39420	MJG (217)	JBVAL-CE C2937
Tolpis barbata (L.) Gaertn.	XX-0-LZ-AD-250-2006	LZ	JBVAL-CE 2808
Tragopogon dubius Scop.	Spain, Valencia, Alzira. *AJMA*	VAL	JBVAL-CE C2938
Tridax trilobata (Cav.) Hemsl.	XX-0-MJG-19--47550	MJG (221)	JBVAL-CE C2939
Trixis californica Kellogg	unknown	MSB (0095990)	JBVAL-CE C2940
Urospermum dalechampii (L.) Scop. ex F.W. Schmidt	Spain, Valencia, Algemesí. *AJMA*	VAL	JBVAL-CE C2941
U. picroides (L.) Scop. ex F.W. Schmidt	Spain, Valencia, Algemesí. *AJMA*	VAL	JBVAL-CE C2942
Ursinia speciosa DC.	XX-0-NCY-20080016G	TEBLI (96)	JBVAL-CE C2943
Vernonia fasciculata Michx.	XX-0-TEBLI-00842	TEBLI (98)	JBVAL-CE C2944
Xanthisma texanum DC.	XX-0-MJG-201301703	MJG (228)	JBVAL-CE C2945
Xanthium italicum Moretti	Spain, Valencia, Algemesí. *AJMA*	VAL	JBVAL-CE C2946
Xeranthemum annuum L.	UKR, Yampilsky, Vinnytsia, Vilshanka river valley. UA-0-MJG-201011234	MJG (231)	JBVAL-CE C2947
